# A new minimally invasive surgical technique for medial retinaculum repair following traumatic patellar dislocation

**DOI:** 10.1007/s00590-017-2120-8

**Published:** 2018-01-04

**Authors:** Haim Shtarker, Michael Assaf, Marshall N. Deltoff

**Affiliations:** 1 Department of Orthopaedics, Galilee Medical Center, 22100 Nahariya, Israel; 2 Unit of Paediatric Orthopaedics, Galilee Medical Center, 22100 Nahariya, Israel; 3 Faculty of Medicine, Bar Ilan University, Tsfat, Israel

**Keywords:** Patella, Trauma, Dislocation, Medial retinaculum

## Abstract

A new surgical method is introduced offering a less invasive approach to reattach the medial retinaculum following acute patellar dislocation. This retrospective analysis comprised 12 cases of medial retinacular repair in 10 patients. The surgical technique achieved reinforced reattachment of the torn region of the medial retinaculum for improved patellar support and stabilization. During follow-up, no recurrent patellar dislocations occurred, except where one patient reported a subjective feeling of patellar dislocation. The average Kujala score for our sample group after 2 years was 89.2. A plethora of methods are described in the literature to repair a tear to the medial patellofemoral ligament, which attaches at the superomedial patella. However, it is our contention that traumatic patellar dislocation invariably results in osteochondral avulsion at the inferomedial patella, refuting medial patellofemoral ligament involvement, and, rather, implicating the inferior aspect of the deep layer of medial retinaculum. Our surgical technique enables stable fixation of the region, decreasing the rate of recurrent dislocations. No grafts are used, permitting tendinous and ligamentous anatomy to remain intact. We further postulate that performing a CT examination preoperatively may reduce time between diagnosis and surgery, in addition to locating fracture sites more precisely.

## Introduction

Acute dislocation of the patella is a common injury which constitutes 2–3% of all knee injuries [1, 2]. Typically, the dislocation is lateral, with the literature reporting rupture of the medial patellofemoral ligament (MPFL) in approximately 90% of cases [3]. Patellar dislocation occurs primarily in adolescents and young adults, especially females, who are active in sports [2, 4], with overall frequency of injury greatest between 10 and 20 years of age [1, 2, 5]. Those under 14 years of age are at highest risk of incurring recurrent dislocation [4]. This injury most frequently occurs via indirect trauma, typically during sports [6], in the form of internal rotation (twisting) of the femur or external tibial rotation while the foot remains stationary [7], or, less commonly, as a result of direct trauma: either via a force applied medially to the patella, forcing it laterally, or trauma to the lateral aspect of the knee, causing valgus stress leading to lateral patellar dislocation.

The most frequent complication of initial dislocation is recurrent dislocation, found in 15–40% of all injuries [8]. Additional complications include injury and functional limitation resulting from chronic instability, patellar and/or femoral osteochondral fractures (approximately 25% of the acute injuries) [2, 9, 10] and complications as a result of the surgery itself, including reduced joint movement, unrestored patellar malalignment, pain and local infection.

Current surgical management includes arthroscopy and a variety of open approaches for repair or reconstruction of the ligaments. While surgical treatment of initial patellar dislocation is associated with a significantly lower risk of recurrent dislocation compared with non-surgical management, there is a higher risk of patellofemoral osteoarthritis [11].

The purpose of this paper is to introduce a new, open, less invasive surgical procedure for reattachment of the medial retinaculum to the patella following acute dislocation. It is our opinion that this method is more mechanically correct. Comparison of the efficacy of this surgical method is made with a variety of other methods presented in the literature. We also propose that use of preoperative CT examination is obligatory, as it enhances the accuracy of locating and identifying the site and nature of the ligamentous tear and any concomitant osteochondral damage.

## Methods

This study is analytical and retrospective. The hospital patient database was used to identify patients who underwent surgery at Galilee Medical Center between 2008 and 2014, utilizing the described surgical technique for repair of a tear of the medial retinaculum due to primary patellar dislocation. Ten patients (a total of 12 knees) were diagnosed and treated for medial retinacular tears between 2008 and 2014. Our study population was 70% male, with ages ranging from 9 to 19 years (average 13.5 years). They were in good general health, with all injuries occurring during sports activity or dancing. 91.7% of the dislocations were due to indirect injury. All dislocations were first-time occurrences; none were recurrent. Patients with comorbid musculoskeletal conditions, e.g., Marfan’s syndrome, or congenital osseous deformities, e.g., osteogenesis imperfecta, severe trochlear dysplasia and valgus deformity of the knee, were excluded from the study. All patients underwent a CT malalignment test; patients with femoral anteversion and/or tibial torsion were also excluded from consideration. One patient, treated for both knees, demonstrated bilateral patella alta, patellar misalignment and pes cavus; another patient had unilateral patella alta.

The most common complaint upon presentation was continuous severe pain (75%), followed by restriction/limitation of movement (33.3%), swelling (16.7%) and episodic severe pain (16.7%). Intra-articular bleeding accompanied the patellar/retinacular injury in 50% of cases.

Closed reduction of the patellar dislocation without general anesthesia was performed in the ER in all cases, after the patient was calmed down or sedated in order to permit the procedure. All patients underwent preoperative clinical examination and imaging.

**Fig. 1:**
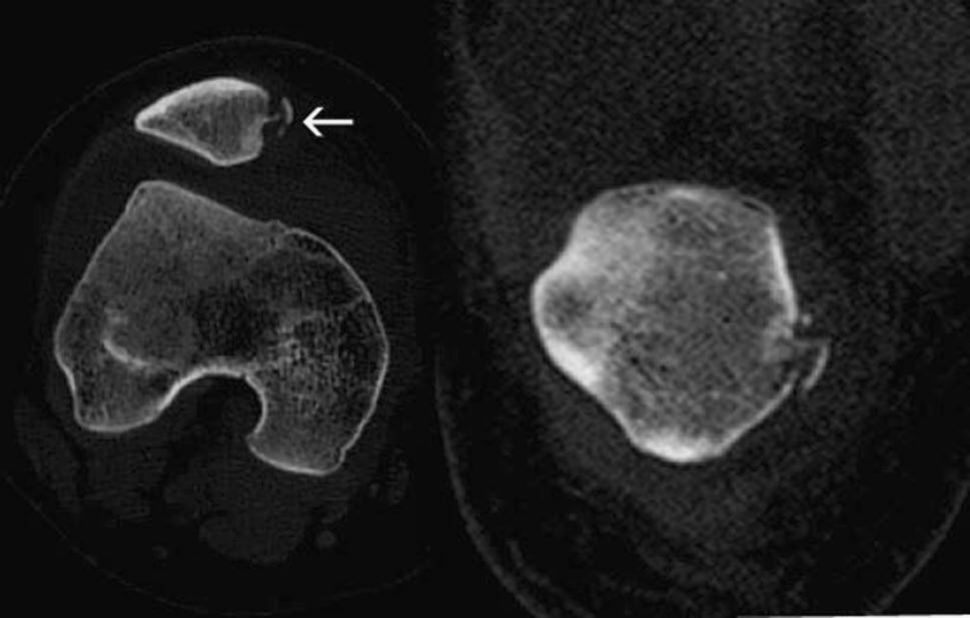
CT of dislocated right patella demonstrates lateral translation of the patella out of the trochlear notch, as well as the comminuted fracture of its medial aspect (arrow). The frontal image (on right) establishes that the location of the avulsion is not superomedial, but rather in the inferior half of the medial patella, mitigating against medial patellofemoral ligament involvement

A major issue in a successful treatment is identifying the avulsion fracture accompanying the retinacular tear after the initial event of dislocation. We performed low-dosage CT prior to surgery in order to increase the efficacy of diagnosing any avulsion fractures of the patella (Fig. 1) and to assess for existing osseous risk factors to dislocation such as patellofemoral joint malalignment, patellar tilt, patellar translation, tibial tuberosity—trochlear groove (TT-TG) distance > 20 mm, tibial tubercle malalignment and trochlear dysplasia [2]. In addition, the CT exam is efficient in identifying rotational deformities of the long bones [2]. As avulsion fractures were diagnosed in 100% of our patient population, all were referred for surgery. The timeframe from initial diagnosis to surgery averaged 7.5 days.

With the patient supine, under general anesthesia, a stability test of the patellar–trochlear track was performed, as well as a provocative test for dislocation (surgeon pushing patella laterally). A tourniquet was applied to the lower limb as high up on the thigh as possible. The lower limb was then scrubbed. A 4–5 cm longitudinal incision was made in the patellar midline, from the upper pole to the lower pole.

**Fig. 2:**
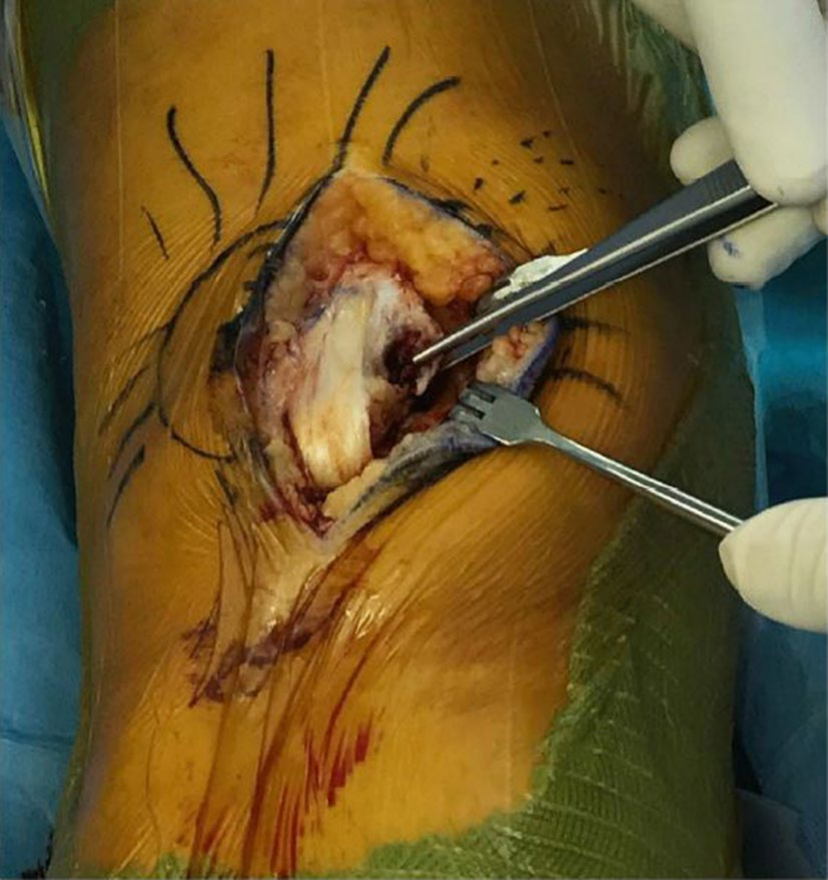
Identifying and isolating the avulsed fracture fragments. Note the location of fragments at the mid-inferior patella, not at the superomedial aspect

Approximately 1 cm from the medial rim of the patella, dissection laterally of a portion of the patellar periosteum was undertaken in order to prepare a lateral tissue flap. The fragments of the avulsion fracture attached to the deeper layer of the medial retinaculum were then identified (Fig. 2). Typically, the fragment or fragments are quite small, only 1–3 mm in width, but are easily recognizable. Evacuation of the hemarthrosis was performed, followed by careful washing of the joint and cleaning of the fracture site with curette; the avulsed bone fragments were then removed. The knee was examined internally for any additional injury.

**Fig. 3:**
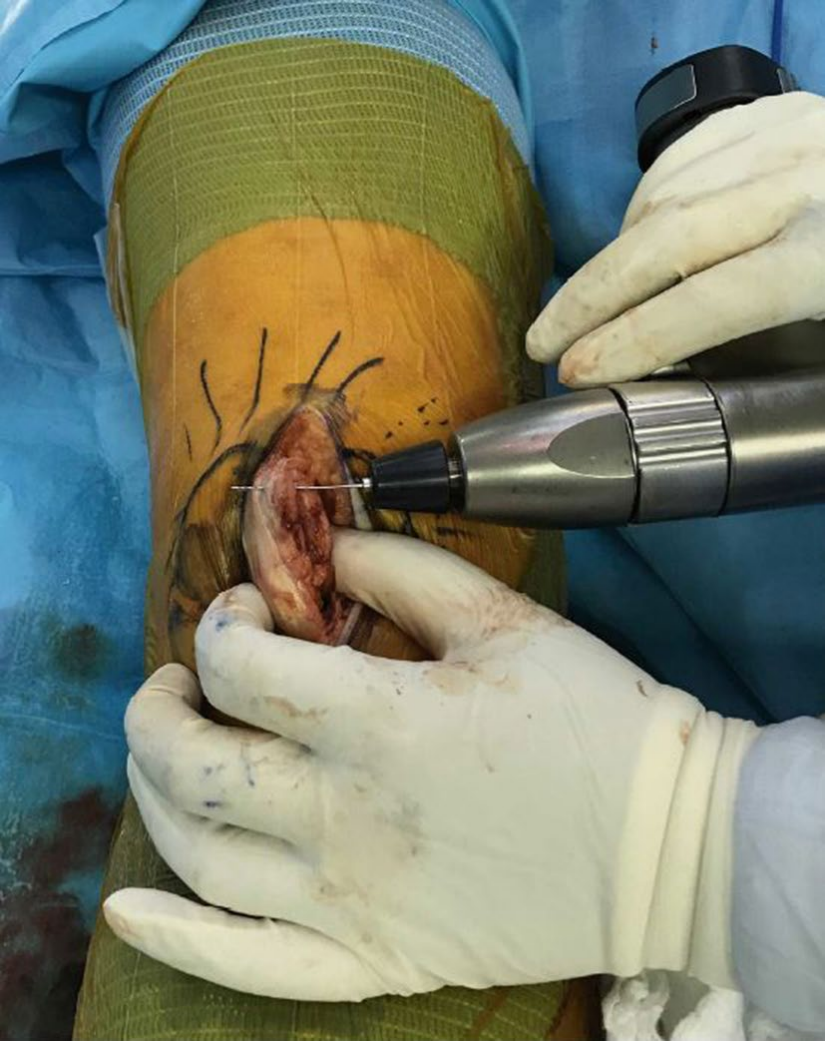
Drilling tunnels from medial patellar slot through to anterior surface

A slot, 3–4 mm in depth, was prepared in the medial rim of the patella using a small-size oscillating saw; the slot was positioned so as not to include any of the inferior patellar cartilage. The size of the slot was proportional to the size of the avulsed portion of the medial retinaculum. Patellar tracking was then checked in order to assess the dynamic relationship of the patella to the trochlear groove. In cases of maltracking, or a tight lateral retinaculum, at this stage of the surgery, lateral release of the patella was performed utilizing diathermy (electrocauterization) under direct vision using a fiber optic light source. An additional check of the patellar tracking was done at this point. A minimum of 4 tunnels were then drilled through the patella, from the deepest point in the surgical slot anteriorly to the patellar surface, in order to permit suturing for deep medial retinaculum reattachment (Fig. 3). If the patellar size permits, and the avulsion is wide, it is possible to drill additional tunnels in order to allow for more sutures. It is important, however, that the holes should not weaken the medial patellar rim. The drill size was maximum of 1.5 mm, in order not to weaken the bone.

**Fig. 4:**
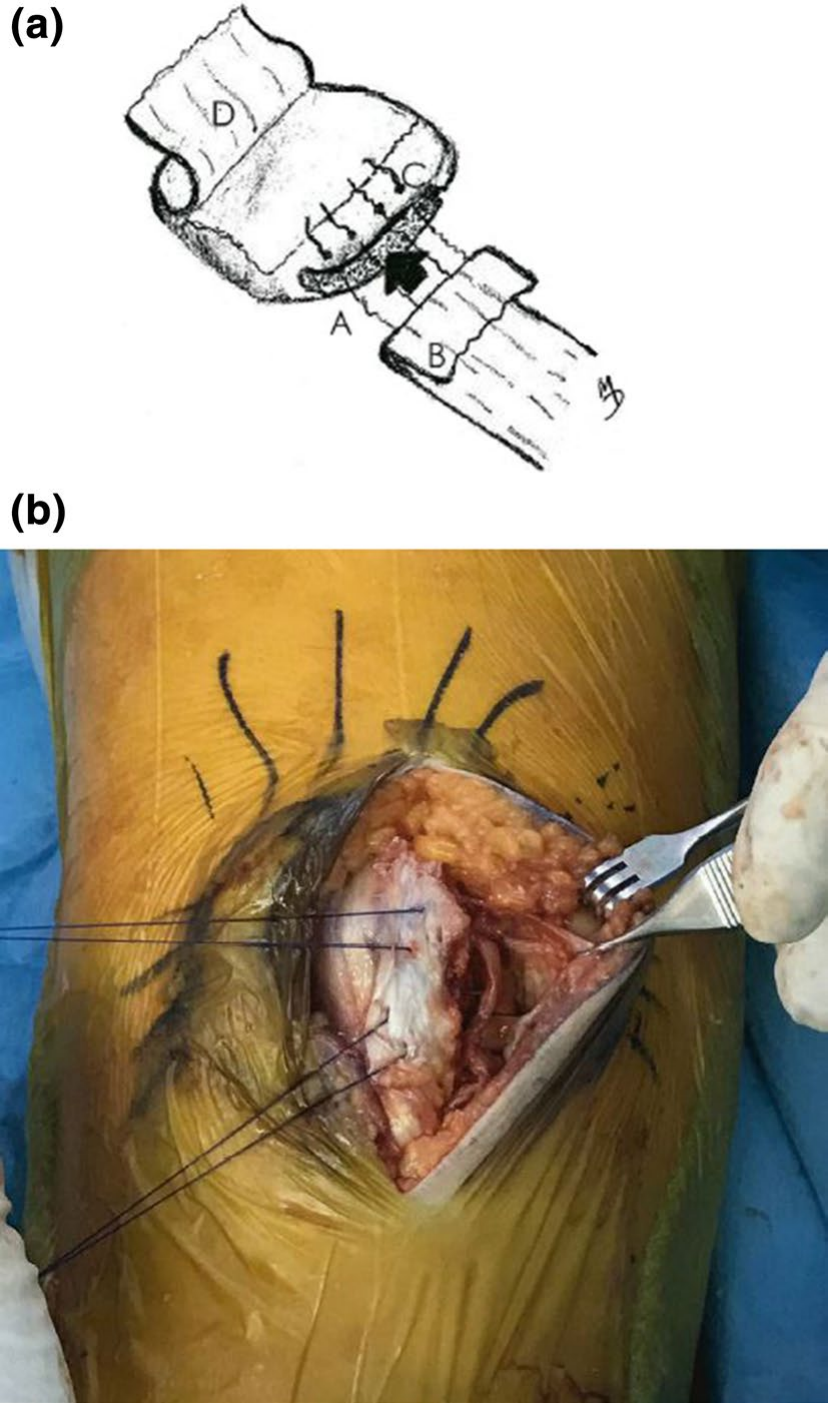
a Right patella. A slot (A) is prepared in the medial rim. Avulsed portion of the deep medial retinaculum (B) is folded over and drawn into the slot, then sutured to the patella via the drilled tunnels (C). The anterior patellar periosteum (D) has been peeled back to prepare a flap for later use. **b** Sutures are then drawn through the tunnels, securing the deep medial retinaculum into the patellar slot

At this point, 00 gauge of PDS suture was passed through the tunnels and the deep layer of the retinaculum. It is imperative that the sutures be located at least 8–10 mm from the edge of the torn deep retinaculum in order to allow for the creation of a protruding tissue flap (Fig. 4).

**Fig. 5:**
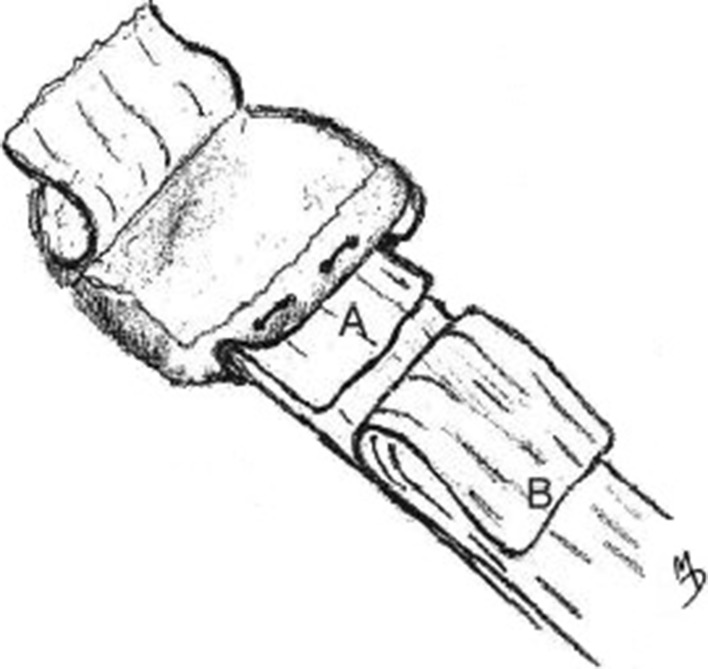
Deep layer of medial retinaculum (A) sutured into slot, with protruding flap. (B) Folded-back superficial layer of medial retinaculum

**Fig. 6:**
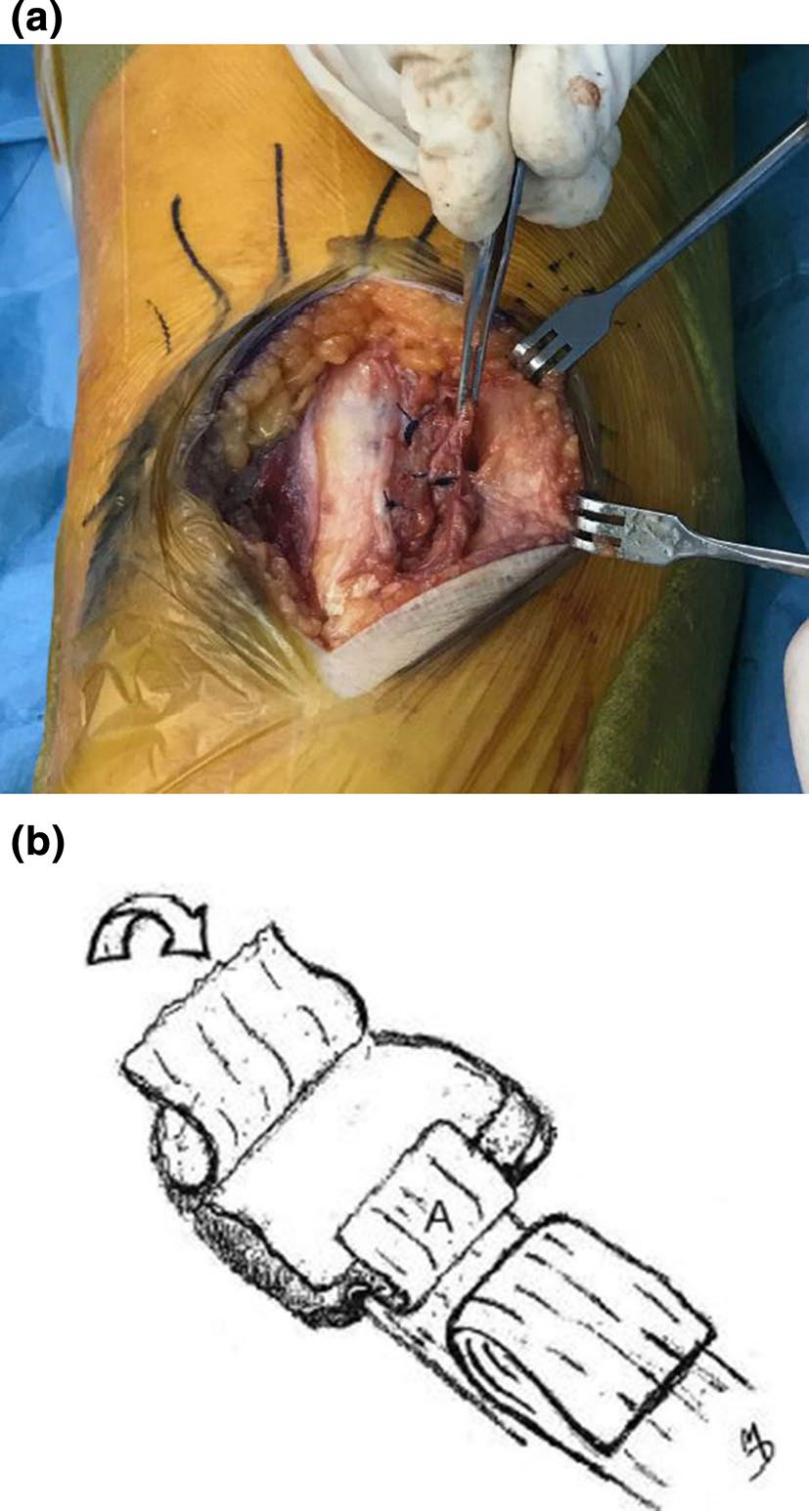
a Preparing to fold the protruding flap of deep layer of medial retinaculum over the anterior patella. **b** Protruding flap of deep layer of medial retinaculum (A) is now folded over and sutured to medial periosteum on anterior patella

**Fig. 7:**
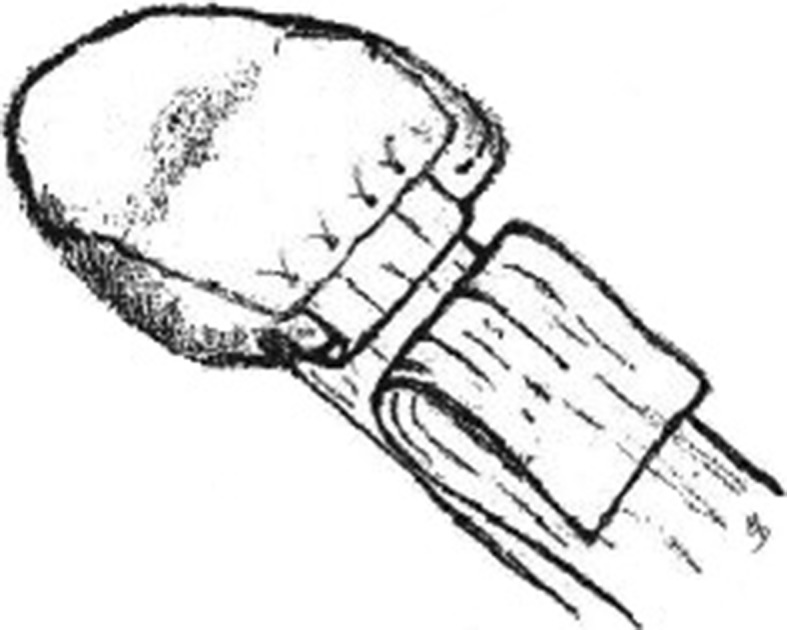
Previously prepared periosteal flap is placed back down and also secured to the anterior patella, covering the flap of the deep layer of medial retinaculum

**Fig. 8:**
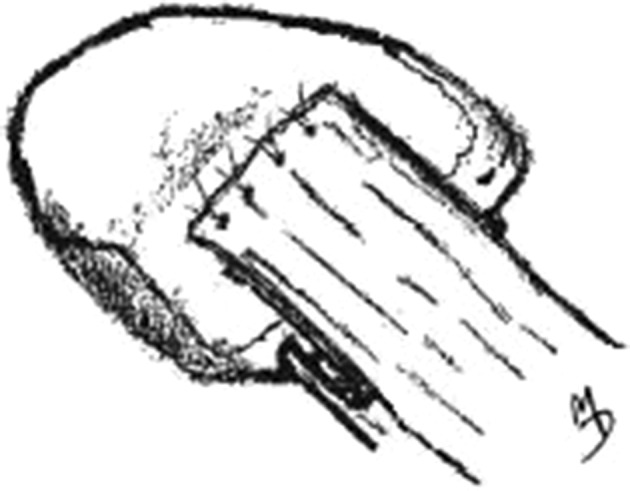
Superficial layer of medial retinaculum is then folded over to form the final covering and sutured to the patellar periosteum

The portion of the deep retinacular layer containing the sutures was then drawn into the slot and secured using the previously drilled holes (Fig. 5). Next, the flap described above, consisting of the remaining edge of deep retinaculum, was folded back over the anterior patella and sutured to the periosteum (Fig. 6), The lateral periosteal flap prepared earlier was then replaced over the anterior patella, so that its edge now overlapped the deep medial retinacular flap; it also was then sutured securely (Fig. 7). Lastly, the superficial layer of the medial retinaculum was overlapped over all other layers on the anterior patella, forming a final covering layer, and was securely sutured in place (Fig. 8).

Suturing of subcutaneous fat, followed by intracutaneous suturing, was then completed with resorbable sutures. No drainage hemovac to the joint was used. A sterile dressing was applied, and the knee was placed in a molded Tutor cast. 91.7% of the patients were put into a cast only; the additional patient was first casted and then put into a dynamic Sauter cast. Duration of surgery, on average, was 76.5 min (range: 54–104 min, not including time of induction and anesthesia). The average duration of hospitalization following surgery was 2.5 days. One week following surgery, a window may be opened in the cast to check the operative wound.

The postoperative protocol began with full weight-bearing on the day following surgery. Active straight leg exercises and active ankle range of motion exercises are introduced and encouraged from the first-day post-surgery. The patient wears the Tutor cast for the first 4 weeks; this is replaced with an Orliman brace for the next 2 weeks. The brace is initially fixed in 180 degrees of extension; every 2 weeks, an additional 30 degrees of flexion is added. The brace is discontinued when the patient has achieved 90 degrees of knee flexion.

## Results

Patients were requested to attend follow-up 2 weeks after surgery, then again after 4 weeks, 6 weeks, 2.5 months, 1 and 2 years. Outpatient clinic follow-up included clinical examination and X-rays. In addition, at 2 years, the Anterior Knee Pain Scale (Kujala) questionnaire was administered, in order to subjectively assess knee function. This is an accepted timespan for assessing final functionality of the knee (Dr. Urho Kujala, personal correspondence e-mail, November 2, 2016). Continuing follow-up was at the discretion of the treating doctor. Clinical assessment at initial follow-up appointment, 2 weeks post-surgery, revealed instability in only one knee and some restriction of motion in 5/12 knees (41.7%). None of the patients demonstrated swelling or intra-articular bleeding.

At the half-year mark, none of the patients demonstrated or complained about any swelling or instability. Only two patients had any restriction of range of motion at one half year post-surgery; flexion was reduced from 130° to 110°. After 1 year post-surgery, only one of the patients still demonstrated the restricted flexion. Also after 1 year, one patient reported a “feeling” of a recurrent dislocation, with no positive clinical examination findings, including a negative patellar apprehension test. At the 2-year follow-up, the Anterior Knee Pain Scale (Kujala) questionnaire was administered; the resultant mean score was 89.20, with a range of 78.95–99.45. There was no statistical difference with the results in the literature (binomial test, one sided, *p* = 0.284). Inquiry of the patients at the 2-year follow-up assessed ability to run, walk, and if there was any recurrence of dislocation. These results are summarized in Table 1. There was no occurrence of osteoarthritis noted in any patient on radiographic follow-up.

**Table 1:** Condition of knee at 2-year follow-up as reported by patient

Knee function after surgery at initial follow-up	Number of knees	
*Running after surgery*		
Without difficulty	7	58.3%
Pain after > 2 km	2	16.7%
Unable	3	25.0%
*Walking after surgery*		
Without difficulty	8	66.7%
Pain after > 2 km	1	8.3%
Pain after 1–2 km	3	25.0%
*Recurrent dislocation at initial follow-up*		
No dislocation	11	91.7%
At least 1 dislocation after surgery (feeling or sensation)	1	8.3%

## Discussion

This study concludes that the majority of damage to the medial holding elements of the knee following acute traumatic patellar dislocation occurs to the medial retinaculum rather than the MPFL. All avulsions of osteochondral fragments in our patient population occurred at the inferomedial aspect of the patella, anatomic site of medial retinaculum attachment, not at the superomedial patella, the site of insertion of the MPFL.

Our utilization of low-dosage CT prior to surgery achieved a high degree of accuracy in identifying the point of retinacular rupture and the accompanying osteochondral fracture and its fragments. CT is more readily available and less expensive than MRI, enabling optimal visualization of osseous structures and relationships versus other imaging modalities. Stanciu et al. [12] found that CT imaging is 1.5 times more sensitive in locating irregularities in patellar tracking than standard radiographic imaging. Among other advantages of CT are the absence of structural overlap or image distortion, the ability to obtain axial images of the patellofemoral joint at angles less than 20 degrees of knee flexion for enhancing assessment of subluxation, and noting and measuring tibial tubercle lateralization, to better identify patients with patellofemoral malalignment [13]. Of particular note in the literature is that the presence of osteochondral fractures was missed in approximately 30–40% of the X-rays performed at initial assessment [2, 14, 15].

Surgery to reattach the medial retinaculum to the patella should result in a decrease in frequency of recurrent patellar dislocation and prevent symptoms of patellofemoral instability, while facilitating as normal a return to pre-injury activity as possible.

The functional evaluation was examined at follow-up at ½, 1 and 2 years following surgery, with Kujala scoring at 2 years. Functional evaluation at initial follow-up revealed that 66.7% went back to walking without difficulty and 58.3% went back to running with no difficulty. These results are also similar to data reported in current literature [16–19]. However, by contrast, in a study by Atkin et al. [6], conservative treatment resulted in restriction of sports activities in 58% of patients after 6 months.

Administration of the Kujala questionnaire at the 2-year mark in our study demonstrated an average score of 89.20 (range: 78.95–99.45). This result compares favorably with the literature, which reports similar scores 2–2.5 years post-surgery of 73.40 [19], 80.90 [20], 86.00 [15], 87.00 [14], 89.20 [21], 91.40 [22], 92.00 [23], 94.03 [24], 94.69 [16] and 99.69 [18].

Our present study did not demonstrate any incidence of recurrent dislocation 2 years following surgery; this is identical with the findings utilizing several other techniques [16, 18, 19, 22–25]. However, the literature does mention various percentages of recurring dislocation following certain surgeries [1, 15, 17, 20, 21, 26, 27].

In summary, the described surgical approach utilized at our institution resulted in stable fixation of the attachment of the ruptured medial retinaculum to the patella with no objectively demonstrable recurrence of patellar dislocation during follow-up for 2 years after surgery.

The surgery is safe and maintains all ligamentous and tendinous anatomy; no graft tissue is harvested. The value of performing diagnostic CT was also noted in order to better isolate the site of tear and the planned surgical treatment. We conclude that our surgical method of medial retinaculum reattachment achieved excellent functional results, comparable to other existing techniques, both open and arthroscopic.

The primary limitation of our study is patient sample size. The number of patients who underwent surgery in the department due to patellar dislocation during the period examined, was low and stood at only 10 (12 knees). In addition, our study is retrospective and there was no control group. Following the promising results of this preliminary study, it is recommended that a further study with larger patient base be considered.
